# The Medical Diseases of Egypt

**Published:** 1906-06

**Authors:** 


**The Medical Diseases of Egypt.** By F. M. SANDWITH, M.D., F.R.C.P. Part I. Pp. vi., 316. London: Henry Kimpton. 1905.

This work is in part a text-book for the Egyptian student of medicine, and as such deals in the present volume with the common infectious diseases, such as scarlet fever, measles, &c. Details of the course and symptoms of these diseases are not supplied, the author assuming that these are obtained from some hand-book on general medicine. The subject of treatment is often dismissed in a single line, as under scarlet fever: "Treatment—Urotropin may be given during the third week if scarlatinal nephritis be feared."

Dr. Sandwith is at his best when dealing with the history and geographical distribution of each disease in Egypt, and when dealing with such diseases as bilharziosis, ankylostomiasis, and pellagra. It is interesting to note that he favours the view that the parasite may enter the system by penetrating the skin, not only in ankylostomiasis, but also in bilharziosis. The work will be appreciated by English doctors visiting or residing in Egypt.

